# 1019. The Future is Embedded in the Present - Characterizing Pharmacist Interventions in an Infectious Diseases Clinic

**DOI:** 10.1093/ofid/ofac492.860

**Published:** 2022-12-15

**Authors:** Shawn Depcinski, Ashish Bhargava

**Affiliations:** Ascension St. John Hospital (Center for Internal Medicine), Grosse Pointe Woods, Michigan; Ascension St. John Hospital; Wayne State University School of Medicine, Grosse Pointe Woods, Michigan

## Abstract

**Background:**

Incorporating clinical pharmacists into the outpatient care setting can improve patient outcomes; however, the focus has mostly been on primary care. In 2021, Ascension Specialty Pharmacy embedded clinical pharmacists into clinics prescribing specialty medications to increase prescription capture and improve patient care. The clinical pharmacist incorporated into the Ascension St. John Infectious Diseases clinic provides patient outreach, care coordination, financial and prior authorization assistance, medication reconciliation and counseling, and clinical interventions. This study characterizes the pharmacist interventions provided during a six month timeframe.

**Methods:**

As of September 2021, the clinical pharmacist’s interventions were consistently captured in a documentation system. Each patient encounter that resulted in one or more interventions were entered as one documentation event, which captures type of problem, recommendation category, intervention acceptance, and estimated time spent. If more than one problem within the same type occurred on a patient, it could only be recorded as one problem. A report was pulled over a six month period of September 2021 through February 2022.

**Results:**

During the timeframe reported, infectious disease specialty prescriptions filled at Ascension Specialty Pharmacy had nearly doubled from before embedding the pharmacist (monthly average, 115 vs. 66). During the study period, 316 patient encounters resulted in clinical pharmacist interventions, which included 594 problem types. The frequency of problem types included under-treatment (32%), medication monitoring needed (28%), suboptimal drug (18%), sub-optimal dosing/duration/frequency/administration (12.52%), nonadherence (5.92%), and adverse drug event (4.74%). Pharmacist interventions were accepted 90% by physicians and 81% by patients. The pharmacist time spent on interventions was estimated at 203 hours during the study period.

**Conclusion:**

Despite limitations in how interventions are documented and reported, this study demonstrates the significant impact a clinical pharmacist embedded into an infectious diseases clinic can have on optimizing patient care.

**Disclosures:**

**Shawn Depcinski, PharmD**, bioMérieux: Honoraria.

